# An Improved Method of Performing the Radical Operation in Empyema

**Published:** 1877-06

**Authors:** E. Fletcher Ingals

**Affiliations:** Lecturer on Diseases of the Chest and Physical Diagnosis in Rush Medical College


### AN IMPROVED METHOD OF PERFORMING THE RADICAL OPERATION IN EMPYEMA.

By E. Fletcher Ingals, M. D.

(Lecturer on Diseases of the Chest and Physical Diagnosis in Rush Medical College.)

The removal of pus from the pleural sac by means of the aspirator, even though often repeated, is not likely to cure the patient, however, in rare instances recovery follows this method of treatment, therefore it is advisable to give it a trial.

As soon, however, as it becomes apparent that this procedure is not sufficient, the radical operation, or the establishment of an opening for free drainage and cleansing of the cavity is indicated.

With regard to the operation, Prof. Flint in his work on practice, says, " An opening should then be made at the bottom of the pleural sac, allowing the pus to escape freely. Air will gain admission into the chest, and, since this is unavoidable, its ingress and egress should be unobstructed. A small orifice, not sufficient for the free escape of the pus, and allowing air to be pent up within the pleural cavity, is injudicious. The pus becoming decomposed and fetid, acts as an irritant on the inflamed surfaces, whereas, if it drain away as fast as it is formed, and the cavity of the pleura be well ventilated, this result does not follow."

Dr. W. W. Gerhard, in the revised edition of his work (1860), remarks of this operation, the chances of recovery are not on the whole increased by the operation, unless it be performed very carefully, and it is one which we should not practice, unless it be to relieve excessive dyspnœa. After refering to Dr. Bowditch's method of operating, he says, "I must confess that the operation is always of doubtful benefit, and very often inadmissible. Indeed, in ordinary cases of chronic pleurisy, I would not dream of performing it."

On this subject, Prof. Frank H. Hamilton in his work on military surgery, says, "The existence of pyothorax having been fully ascertained, no time ought to be lost in giving the pus a thorough evacuation." He also says, regarding idiopathic pyothorax—this term being employed in contradistinction to traumatic—"It is of the highest importance to exclude air." On the contrary, Doctor Fuller thought the admission of air of not the slightest importance.

Doctor Bowditch is in favor of excluding the air, though he does not lay great stress upon this point. The use of a drainage tube as first proposed by Chassaignac, and afterwards practiced by Drs. Gosselin, Goodfellow and DeMorgan, is recommended by Drs. Waters, Watson, Aitken and others. Prof. Gross in his System of Surgery, fourth edition, 1866, says of the use of the drainage tube, "This treatment it seems to me, should not be encouraged, as it is both harsh and dangerous." The drainage tube here mentioned was introduced between the fifth and sixth ribs and brought out at the lower part of the pleural sac through a counter opening.

Fraentzel in the fourth volume of Ziemssen's Cyclopædia, says of this treatment: "No physician would now carry out drainage in the way it was proposed by Chassaignac."

In the Boston *Medical and Surgical Journal*, August, 1874, Dr. Henry Clarke, of Worcester, after reviewing the various methods of operating that have been suggested, remarks, that after two thousand years of experience and prolonged discussion, concerning the expediency of the operation and the method of operating, we come back substantially so far as relates to empyema, to the simple practice of Hippocrates, that is to opening freely with the knife and keep the incision open so long as pus continues to collect.

The weight of authority is in favor of free drainage, and while many do not think the entrance of air injurious, perhaps for the reason given by Dr. Flint, viz., that it is unavoidable, there are many others who think its exclusion a matter of no little importance. To accomplish this end, it has been recommended that the operation be made under water with the patient in a bath, and that the wound be hermetically sealed.

Mr. Erichsen suggests that a canula or an elastic tube may be left in the wound to allow the pus to be drawn off and the cavity cleansed. The late Prof. Freer, Drs. Andrews, Powell, and others have used a siphon drainage tube introduced through a free opening into the chest; but all the methods heretofore recommended, contemplate either the frequent repetition of the operation or the admission of air.

Frequent operations by means of the aspirator have not thus far been attended with satisfactory results, and although the entrance of air into the pleural sac is not a serious accident, still it leads to putrefactive changes in the pus, if this be not removed so fast as formed; and, from several cases observed in the Roosevelt Hospital in 1873, it appears that the amount of pus secreted is much greater in patients where air is freely admitted than in those where it is excluded, another fact in support of the position of Drs. Hamilton, Bowditch and others.

By the modification in the operation suggested in this paper, we secure the essential points insisted upon by nearly all who have written on this subject; viz., free drainage and the exclusion of air.

For the improved operation the following articles are required: A trochar, the canula of which is of just sufficient size to allow the passage through it of a drainage tube; a piece of soft rubber for a valve, 2½ inches in diameter, such as dentists use for the rubber dam, excepting that it should be about twice as thick; a piece of rubber tubing (drainage tube) about 3-16 of an inch in diameter, and a foot and a half to five feet in length; an elastic bandage, two inches in width, long enough to reach around the patient's chest and pin; and a small ring, which, when slipped over the bent drainage tube will hermetically seal it at the point of flexure.

The drainage tube should be perforated on the sides in three or four places, near the extremity, which is to be inserted into the chest. It should then be marked with ink at distances of about six and nine inches from the same end, so that when introduced, we may know how much of the tube is within the pleural sac.

The operation is performed in the following manner: The patient being ready, the tube is filled with water, its distal end closed by the ring, and placed in a basin of water beside the bed. The trochar is thrust through the centre of the small piece of rubber, which is to act as a valve, and this is drawn over the canula. Having oiled the trochar, it is to be thrust into the chest, at either of the points usually recommended for the operation.

As the stilet is withdrawn, the thumb should be placed over the end of the canula to stop the flow of pus until the drainage tube can be reached. The tube is then crowded through the canula for a sufficient distance, and the canula is withdrawn; as it leaves the chest walls, the rubber valve is slipped off upon the tube close to the surface of the chest. The extremity of the tube may now be opened under water, the canula slipped off and the pus allowed to flow.

As the stilet is withdrawn, the pus escapes in a full stream, and during the passage of the tube through the canula no air can enter, on account of the pressure of pus from within.

In this way, the operation can easily be completed without the entrance of a single bubble of air.

After the tube is inserted, an aspirator can be attached, or the pus may simply flow out through the siphon. The latter method is sometimes preferable, as the aspirator is liable to cause too great atmospheric pressure upon the expanding lung, and consequent rupture of some of the smaller blood vessels. After the pus has ceased flowing, the cavity may be washed out with a one or two per cent. solution of carbolic acid, at 100.° F., or the cleansing may be deferred for twenty-four or forty-eight hours.

The latter method would probably cause less irritation than the former.

After the pus has been discharged, a slit should be made in the middle of the elastic bandage, a couple of inches in length, for the passage of the tube, and the bandage should then be pinned snugly about the chest, over the rubber valve.

In the after treatment, the pleural sac should be cleansed once or twice daily with a weak solution of carbolic acid. During the treatment, if the cavity ceases to diminish in size, other injections may be substituted.

In cleansing the cavity, the end of the tube should be placed under water, in a basin, which can be raised to any desired height. The fluid flows into the chest through the siphon and then out by the same means.

Between the visits of the physician, the end of the tube may be left under water, to which a little carbolic acid has been added, or it may simply be bent upon itself and closed, by slipping over it the little ring. Though the former method is preferable, the latter will sometimes be found necessary with restless patients. If the latter plan is adopted, the cavity should be cleansed twice a day.

For two or three days, the soft tissues in the intercostal space grasp the tube firmly and keep the cavity hermetically closed, but at the end of this time, the tube becomes loose in the opening through the chest walls, so that air would enter, or the tube would fall out, were it not for the piece of rubber, which was placed on it when the canula was withdrawn.

This rubber not only acts as a perfect valve, when held by the elastic band, to prevent the ingress of air, but it also holds the tube in position, with sufficient firmness to prevent its being withdrawn by any ordinary force. The tube may be still further secured, if thought best, by a bandage placed about the chest.

The slit in the elastic should be long enough to allow some movement of the bandage without traction on the tube.

The force with which the fluid flows into the cavity may be perfectly regulated by the height of the column of water in the siphon. As soon as the cavity becomes slighty distended, the patient will complain of uneasiness or pain in the chest, and then the tube should be at once lowered, to stop the flow.

As healing progresses, the size of the cavity may be accurately determined from day to day by the amount of water required to fill it.

This has the advantage over methods usually recommended of occasioning but a trifling wound, and consequently slight constitutional disturbance from that cause—of preventing the entrance of air—of simplicity in the operation and convenience in after treatment—of painlessness after the soreness from the puncture has subsided, and of comfort and neatness for the patient. It is nearly as simple as paracentesis with the aspirator, and is attended with little if any more danger to the patient.

It might be used in those unyielding cases of chronic pleurisy with serous effusion, where the cavity rapidly refills after paracentesis.

In illustration, I subjoin the reports of two cases operated upon by this method.

Case I. A patient of Dr. P. H. Matthei. Seen in consultation with Drs. Matthei and E. Ingals, December 1st, 1876. E. G., aged four years, was attacked with rheumatism four weeks ago. A few days later complained of pain in left side, evidently of a pleuritic character. To-day the patient complains of severe pain in left side of neck; he has lost considerably in strength and weight,—appetite poor, thirst considerable, digestion fair, bowels loose, urine scanty at times and profuse at others. Pulse small and quick, 130 per minute; respirations, 40. Frequent hacking cough with no expectoration. Curvature of spine with immobility of left side and bulging, especially of left mammary region. Flatness over greater part of left side, with feebly transmitted respiratory sounds. Apex beat of heart within half an inch of the right nipple. Just below the nipple the left side measured ten and five-eighths and the right nine and five-eighths inches. Introduced needle of aspirator near the angles between the eighth and ninth ribs, and withdrew five and a half ounces of thick yellow pus, when coughing came on and the pus ceased flowing; although the cavity was not empty the needle was withdrawn. After the operation the apex beat could be felt about three-eights of an inch nearer the sternum. Soon the child became quiet, and he subsequently did well for several days. The severe pain in the left side of the neck had been entirely removed by the operation.

Dec. 7th. Pain in neck has returned. The patient very restless and suffering from dyspnœa. Punctured the chest again near the same point as before and withdrew eighteen ounces of healthy pus. A few drachms of blood flowed with the last of the pus. The impulse of the heart changed in consequence of operation from a position near the right nipple to the left side of the sternum. The respiratory sounds became quite distinct in left lung. The child did well for two or three days after the operation.

Dec. 12th. Respirations, 44; pulse, 150. Patient very weak. Apex beat near right nipple. Performed paracentesis near the point of the last puncture and withdrew fifteen and a half ounces of pus. The heart returned nearly to its normal position and the left side which had measured eleven and one-fourth inches before the operation, measured only ten and three-fourths afterward.

Dec. 21st. The patient did well for four or five days after the last operation, but for a few days he has been losing; suffering greatly from dyspnœa and pain. During last night he took, beside several grains of bromide of potassium and several drops of laudanum, a grain and a half of morphia in doses of a quarter of a grain each, yet he did not sleep. There is œdema of the left side of the chest as low as the last rib where it abruptly terminates. The struggles of the child in opposing another operation, caused the wound of the last puncture to re-open. From the opening, thick pus slowly escaped.

In the afternoon of this day, I operated in the method described, excepting that air was allowed to enter the chest. The aspirator was applied after the dressings had been secured and the air withdrawn with the remaining pus. Twenty-three ounces of a thick, greenish-yellow pus were obtained. About half an ounce of blood flowed after the aspirator had been attached. The pleural sac was not cleansed in this case, until the following day, afterward it was washed out every day with a one per cent. solution of carbolic acid. The tube was bent upon itself and kept closed, excepting morning and evening when it was opened under water and the pus allowed to flow out. The pus remained more or less bloody for several days. The cavity was rapidly obliterated so that in two weeks it would hold only two ounces. Three weeks after the operation it was noted that the child had been suffering for several days with rheumatic pains, and that he had just passed through an attack of varicella. Not more than half a drachm of pus had escaped morning and evening for several days, and the cavity would not hold more than two drachms.

Jan. 13th. The bandage having been removed, the tube fell or was crowded out to-day—cavity entirely obliterated.

Four weeks after the operation, the patient was discharged by Dr. Matthei as cured; the opening in chest was healed, the lung expanded freely, the impulse of the heart was normal and there was no dyspnœa. A month later there was slight curvature of the spine and depression of the left side, but after a few weeks the child presented a normal appearance of the chest and was in perfect health. During the treatment from two to three grains of quinine were administered daily, and Dover's powder or morphia were administered when needed to relieve pain and secure rest.

March 18th, 1877. Case II. Mrs. P., aged 30, mother of two children, youngest about three years of age. Has been sick about eighteen months. From the history, seems first to have had pleurisy. Was attended for several months by regular physicans, and then fell into the hands of a quack, who maltreated her for nine months.

The patient is very feeble; harassed by an almost constant cough, and frequently expectorates a considerable quantity of grayish white sputa, of a nummular form. Complains of severe pain in the right side, for which she is obliged to take frequent doses of morphia. Pulse, 118; respirations, 28.

Dullness on right side above fifth rib in front, and above sixth behind. Flatness below these points, and extending one inch higher in axillary region. When lying upon left side a slight degree of resonance extends about an inch lower in the axillary region. Heart one half inch farther to the left than normal. Retraction of right side. On a level with the sixth costal cartilage the sides each measure nearly fourteen inches, the left may be an eighth of an inch smaller. Left side moves half an inch in full inspiration. No expansion of right side. With the assistance of Dr. D. R. Brower, I administered nitrous oxide gas, and punctured the chest between the seventh and eighth ribs, a little in front of their angles, but as no pus was obtained the needle was withdrawn, and another puncture made about an inch nearer the spine, between the sixth and seventh ribs. From this opening we withdrew six ounces of thick yellow pus. The patient steadily improved after the operation for about three weeks. Twenty-four hours after the operation she was able to sleep comfortably without morphia, as her cough and pain had entirely disappeared. The cough returned in about twelve days, but was not severe. Eighteen days after the operation, slightly amphoric resonance and amphoric respiration were present below the sixth rib, one inch external to the line of the right nipple.

Three weeks after the puncture the coughing became frequent, and the pain returned to the right side. The flatness which had greatly diminished after the operation, became greater than before.

April 15th, four weeks after the aspiration, we again gave nitrous oxide gas, and performed the radical operation as above described, evacuating thirteen ounces of thick pus. We used the aspirator in this case to evacuate the last of the pus; though I now think, it would be better to rely simply on the siphon. One or two ounces of blood followed the aspiration. No air entered the chest. After the pus had been evacuated the cavity was washed out with a weak solution—about one per cent. of carbolic acid.

The end of the tube was kept closed for two or three days, excepting when the pleural sac was cleansed in the morning. Afterward it was placed under water, and constant drainage allowed.

The cavity rapidly diminished in size for eight days, when it would hold only one ounce.

On the evening of the 23d of April the patient, in rising from a chair, accidentally caught the tube and pulled it nearly out of the chest. I was sent for, but finding it impossible to re-insert the tube, and thinking the cavity would heal before the opening in the chest closed, I removed the drainage tube and dressed the external opening with a carbolized compress. Five days later the following notes were made: External opening healed. Patient has gained four pounds in twelve days, and feels well.

The patient continued improving for six or eight days, fol lowing the last entry, and then after a hacking cough, which lasted nearly a day, she was taken with pains in both sides of the chest, and after a few hours the cough became violent. For the following three days she expectorated about a pint each day of the same muco-purulent, nummular sputa as before the operation. At this time there were distinct signs of a pulmonary cavity where the amphoric respiration had been observed three weeks previously, but the pleural cavity from which the pus had been withdrawn was entirely healed. The case is still under observation.

